# A reservoir of ‘historical’ antibiotic resistance genes in remote pristine Antarctic soils

**DOI:** 10.1186/s40168-018-0424-5

**Published:** 2018-02-23

**Authors:** Marc W. Van Goethem, Rian Pierneef, Oliver K. I. Bezuidt, Yves Van De Peer, Don A. Cowan, Thulani P. Makhalanyane

**Affiliations:** 1 Centre for Microbial Ecology and Genomics (CMEG), Department of Biochemistry, Genetics and Microbiology, University of Pretoria, Natural Sciences Building 2, Lynnwood Road, Pretoria, 0028 South Africa; 2 Centre for Bioinformatics and Computational Biology, Department of Biochemistry, Genetics and Microbiology, University of Pretoria, Pretoria, South Africa; 3 Department of Plant Biotechnology and Bioinformatics, Ghent University, 9052 Ghent, Belgium; 4 Center for Plant Systems Biology, VIB, 9052 Ghent, Belgium; 5 Bioinformatics Institute Ghent, Ghent University, 9052 Ghent, Belgium

**Keywords:** Antibiotic resistance genes, Soil resistome, Antarctica, Metagenomics

## Abstract

**Background:**

Soil bacteria naturally produce antibiotics as a competitive mechanism, with a concomitant evolution, and exchange by horizontal gene transfer, of a range of antibiotic resistance mechanisms. Surveys of bacterial resistance elements in edaphic systems have originated primarily from human-impacted environments, with relatively little information from remote and pristine environments, where the resistome may comprise the ancestral gene diversity.

**Methods:**

We used shotgun metagenomics to assess antibiotic resistance gene (ARG) distribution in 17 pristine and remote Antarctic surface soils within the undisturbed Mackay Glacier region. We also interrogated the phylogenetic placement of ARGs compared to environmental ARG sequences and tested for the presence of horizontal gene transfer elements flanking ARGs.

**Results:**

In total, 177 naturally occurring ARGs were identified, most of which encoded single or multi-drug efflux pumps. Resistance mechanisms for the inactivation of aminoglycosides, chloramphenicol and β-lactam antibiotics were also common. Gram-negative bacteria harboured most ARGs (71%), with fewer genes from Gram-positive *Actinobacteria* and *Bacilli* (*Firmicutes*) (9%), reflecting the taxonomic composition of the soils. Strikingly, the abundance of ARGs per sample had a strong, negative correlation with species richness (*r* = − 0.49, *P* < 0.05). This result, coupled with a lack of mobile genetic elements flanking ARGs, suggests that these genes are ancient acquisitions of horizontal transfer events.

**Conclusions:**

ARGs in these remote and uncontaminated soils most likely represent functional efficient historical genes that have since been vertically inherited over generations. The historical ARGs in these pristine environments carry a strong phylogenetic signal and form a monophyletic group relative to ARGs from other similar environments.

**Electronic supplementary material:**

The online version of this article (10.1186/s40168-018-0424-5) contains supplementary material, which is available to authorized users.

## Background

Antibiotic production and resistance have ancient origins (~ 2 Gyr: [1, 2]). While the spread of antibiotic resistance is one of the preeminent challenges facing global public health in the twenty-first century, antibiotic resistance mechanisms predate anthropogenic antibiotic use. Currently, efforts to restrict the wide-spread dissemination of antibiotic resistance have focused on increasing the repertoire of antibiotics in clinics and by exploring both the origins and mechanisms of antibiotic resistance in soil environments [3, 4]. Understanding the sources of antibiotic resistance genes (ARGs) in remote natural environments which have minimal anthropogenic input may be of benefit in tracking the evolution of resistant pathogens [5].

The three major mechanisms of bacterial antibiotic resistance are broadly categorised as efflux pumps, resistance mutations and antibiotic inactivation strategies [6, 7]. Drug-specific efflux pumps have narrow ranges of antibiotic export mechanism and are specific to a particular antibiotic (e.g. in the removal of tetracycline [8]), whereas multidrug efflux pumps have broad specificity and confer resistance to multiple antibiotics. Resistance to nalidixic acid (a synthetic quinolone antibiotic) is conferred to cells with a mutated *gyrA* gene, producing a genotype that is recessive to the wild-type [9]. Common drug inactivation mechanisms in soil communities include β-lactamases, which hydrolyse β-lactam antibiotics such as penicillin [10, 11].

Resistance genes are often encoded on the same gene cluster as antibiotic biosynthesis pathway genes [12], conferring resistance in antibiotic-producing species to the products they synthesise [13]. Resistance determinants are acquired through natural selection, such as after exposure to an antibiotic at sub-therapeutic concentrations [14] and through the horizontal transfer of genomic material between species [15]. Some ARGs that are likely to be exchanged between species are typically associated with transposons or other mobile genetic elements (MGE) such as plasmids or integrons [6]. Extracellular DNA harbouring ARGs can persist in soil matrices, by cation binding, for example, facilitating genomic transfer between members of the soil community [16]. Moreover, the persistence of DNA in frigid environments such as permafrost and cold or polar deserts is facilitated by low temperatures that reduce the activity of endoenzymes that degrade cells and their DNA [17].

Soils are environmental reservoirs of ARGs and are sources of resistance in human pathogens [5, 18]. Anthropogenic practices that release antibiotics and/or components of the human microbiome into the environment are significant sources of global ARG proliferation [19, 20]. However, the spectrum of historical (i.e. not of anthropogenic origin) resistance determinants in the soil resistome remains largely unknown, with extreme and remote soil ecosystems particularly underrepresented.

Microbial communities from remote and pristine soils provide a valid genetic resource for exploring the historical evolutionary origins of natural antibiotic resistance from the pre-antibiotic era [6, 21]. Soils with minimum anthropogenic contamination and anthropogenic-induced selection pressures should reflect only the spectrum of natural antibiotics and their cognate resistance mechanisms [1] with little or no genetic impact from selection pressures introduced by the input of the twentieth century synthetic and semi-synthetic antibiotics [22].

Very few studies have explored antibiotic resistance in so-called pristine soils. Soils from a remote Alaskan environment showed abundant β-lactamases [10], while genes encoding resistance to tetracycline and glycopeptide antibiotics were found in ancient permafrost DNA and isolated cave samples contained multiple antibiotic resistance genes for macrolide glycosylation [1, 23]. However, very little is known about the abundance or diversity of ARGs in pristine Antarctic soils, which represent some of the few remaining environmental niches which are essentially undisturbed by human activity. Antibiotic resistance elements have been found in Antarctic seawater [24, 25] and in McMurdo Dry Valley soils [26], although these sites cannot be considered ‘pristine’ due to the on-going and long-term research programs carried out in these regions. Here we used a metagenomic-based approach to identify the natural diversity of antibiotic resistance genes in silico from remote and pristine Antarctic soils of the Mackay Glacier region. These sites have no known exposure to anthropogenic antibiotics, are accessible to a few researchers, and can be validly considered to be ‘antibiotic naïve’.

## Methods

### Sites, sampling and physicochemical analysis

**Fig. 1:**
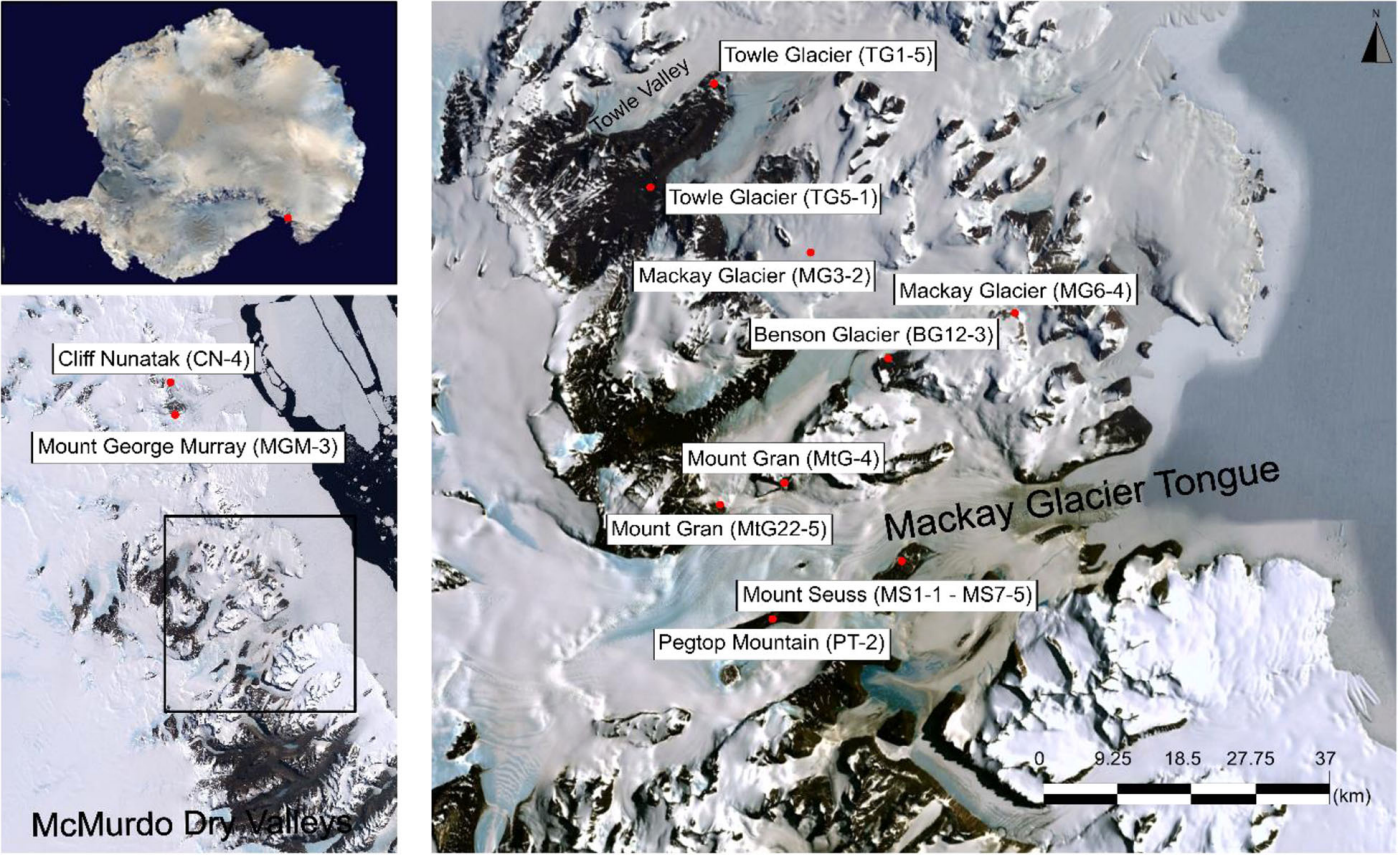
Satellite image of the Mackay Glacier ecotone with the 17 sampling sites indicated. Source: Landsat Image Mosaic of Antarctica (LIMA) Digital Database and Google Earth

Surface soil samples were collected in January 2015 from 17 sites in ice-free areas in the vicinity and to the north of Mackay Glacier, South Victoria Land, Antarctica, which spans ~ 100 km (Fig. 1). Five aliquots of 50 g soil (0–5 cm depth; sieved to 2 mm mesh size) were collected from approximately 1 m^2^ area at each sampling site using sterile methods. Samples were stored in sterile 50 ml polypropylene Falcon tubes (Grenier, Bio-One) at below 0 °C in the field and during transport to the University of Pretoria (South Africa). Soils were analysed for soil pH, total nitrogen, carbon, phosphorus and major cations (K^+^, Na^+^, Ca^2+^ and Mg^2+^) at the Stellenbosch Central Analytical Facilities, Stellenbosch University, South Africa, using standard quality control procedures [27]. Elemental analysis was performed using a LECO TruSpec® Elemental Determinator by combustion analysis. X-ray fluorescence spectrometry for major cations was performed on a Philips PW1404 XRF. Soil pH was measured using 2.5:1 (mass:volume) soil suspensions in deionised water.

### Sample preparation and DNA sequencing

Metagenomic DNA was extracted from each soil sample aliquot in duplicate using an established buffer-chloroform/phenol protocol [28]. Samples with the highest DNA concentration and purity from each site (*n* = 17) were submitted for sequencing at a commercial supplier (MR DNA Lab, Shallowater, TX, USA). Sequencing was performed on a HiSeq 2500 Ultra-High-Throughput Sequencing system (Illumina) using paired-ends (2 × 250 bp) for 500 cycles as per the manufacturers’ instructions.

### Metagenomic assembly and ARG taxonomic identification

Raw reads were quality-filtered, trimmed and screened using Prinseq-lite v0.20.4 [29] in combination with in-house scripts. We used FLASH v1.2.11 (fast length adjustment of short reads) to align high-quality paired-end reads [30]. Sequences were de novo assembled using metaSPAdes v3.9.0 [31], as recommended [32]. The quality of each assembled metagenome (*n* = 17) was assessed using MetaQUAST v4.3 [33]. To provide taxonomic assignments, all contigs were compared to the entire NCBI protein non-redundant database using DIAMOND v0.7.9.58 at an E-value cutoff of 1 × 10^− 5^ [34]. The proportion of species-assigned ARGs at each site was calculated by dividing the number of ARG-containing species by the total number of species present in each community.

### ARG database construction

A local non-redundant ARG database was created by concatenating the Antibiotic Resistance Genes Database (ARDB) [35] and the Comprehensive Antibiotic Resistance Database (CARD) [36]. The concatenated database, *noradab* (non-redundant antibiotic resistance database, is available online (noradab.bi.up.ac.za).

### Resistome identification

Gene prediction for the 17 metagenomes was performed using Prodigal v2.6.3 [37] with the *meta* option specified. Genes predicted by Prodigal were compared against the local *noradab* database by means of BLASTp with an *E* value threshold of 1 × 10^− 6^. Results were filtered for hits with a minimum percentage identity of 75% and alignment length of at least 25 amino acids. For each predicted gene adhering to these parameters, only the hit with the highest score was annotated as an ARG. We used the Markov Cluster (MCL) algorithm to resolve ARG family redundancy [38]. All ARGs were compared against each other in an all-against-all BLASTp approach [39] with a cutoff *E* value of 1 × 10^− 10^. The resulting *E* values were used in the MCL algorithm to cluster ARGs into families with an inflation parameter set to 1.1 and log-transformed *E* values with a limit set at 100. ARG families were annotated according to the corresponding ARDB or CARD database descriptions for all members of the cluster [35, 36].

The relative abundances of ARGs were calculated as the total number of ARGs per sample divided by the total number of Prodigal predicted genes. The relative ARG family abundance was inferred by calculating the number of different ARG families represented by the number of different ARGs in the sample. The local non-redundant ARG database contained 4485 unique AR protein sequences.

### Redundancy analysis

Redundancy analysis was performed in R v3.2.3 (R Foundation for Statistical Computing; https://www.r-project.org) with the *vegan* package (v2.4.0) [40]. ARG presence/absence transformed values and a nine-factor environmental dataset (soil pH, percentage C and N, P (ppm), K^+^ (mg/kg), Na^+^ (cmol(+)/kg), Ca^2+^ (cmol(+)/kg), Mg^2+^ (cmol(+)/kg) and site altitude) were used to evaluate the effect of soil abiotic features on ARG distribution across the environment.

### Mobile genomic elements

To find evidence of mobile genetic elements (MGE) associated with ARGs, we extracted the amino acid sequences from all contigs that were predicted to harbour ARGs. These contigs, together with the peptides identified in all other contigs in the metagenomes, were then compared to the NCBI Conserved Domain Database (CDD)-COG database using reverse PSI-BLAST (RPS-BLAST). For all comparisons, an *E* value of 1 × 10^− 3^ was used to screen for the presence of COGs related to mobile genetic elements [41].

### Network analysis

Co-occurrence networks were produced by obtaining Spearman correlation coefficients (*ρ*) from the bacterial and archaeal relative abundance data for genera present in all 17 communities against all ARGs found in each metagenome. Correlations with rho coefficients greater than *ρ* = 0.6, or below *ρ* = − 0.6, and with significant *P* values (*P* < 0.05) were included in the analysis. Cytoscape v.3.5.1 was used for network visualisation [42].

### Phylogenetic analysis

Phylogenetic analyses were performed using three highly abundant ARG types from three distinct ARG families that were present in at least seven sites. For each site, all predicted proteins identified as the specific ARG and the *noradab* protein with which sequence similarity was ascertained were aligned, together with similarly annotated bacterial protein sequences from a diverse set of soil habitats obtained from the NCBI database online. Multiple sequence alignments were performed with MAFFT [43], and the resulting sequences were trimmed using trimAl [44]. Phylogenetic trees were constructed by RAxML [45] with 1000 bootstraps and automated substitution model selection. A description of the selected ARGs chosen from *noradab* is available in Additional file 1: Table S2.

## Results and discussion

We compiled *noradab* by combining the existing ARDB and CARD databases to remove redundant sequences present in both repositories. ARDB is a widely used and informative collection of ARGs containing 7828 sequences. However, ARDB contains multiple redundant sequences [46, 47], in addition to three sequences in nucleic acid instead of amino acid format [47]. These nucleic acid sequences were removed, and the resulting 7825 protein sequences were inspected for redundancy. We found that 4826 of these sequences shared 100% similarity, as determined previously [47]. The resulting non-redundant ARDB database consisted of 2999 unique protein sequences.

The CARD protein homology sequences (December 2017 release) contain antimicrobial resistance genes but do not include mutations as resistance mechanisms. CARD contains 2169 protein sequences, of which 11 were redundant sequences. Overall, there were 664 shared sequences among the non-redundant ARDB and non-redundant CARD databases. As such, we constructed a non-redundant antibiotic resistance gene database—*noradab*—containing 4493 unique protein sequences with a description or header inclusive of all the descriptions found across all redundant sequences within ARDB or CARD. Clustering resulted in 140 ARG family clusters, which included 47 singletons.

Assembled shotgun metagenomic sequences (contigs) can be used effectively to access full-length antibiotic resistance genes (ARGs) in environmental resistomes [48]. In the 17 assembled Antarctic soil metagenomes, we identified an average of 265,000 open reading frames (ORFs) per metagenome, of which only 177 were annotated as potentially encoding antibiotic resistance. The low levels of ARGs in the soil metagenomes may be due to the stringent selection parameters implemented here (see the “Methods” section) but are more likely to reflect the very low level of anthropogenic impact, and the effective absence of an antibiotic burden, on these soils. The low proportion of ARGs (c.f., total ORFs) is comparable to previous estimates of resistance genes in paddy field soils [49], temperate soils [50] and glacial cores and surface snow samples [21]. Globally, hot and cold desert soil metagenomes are characterised by a lower proportion of ARGs (1.5% of annotated reads) compared to temperate metagenomes (4.8%), which is thought to indicate reduced competition between members of desert soil communities [51].

**Fig. 2:**
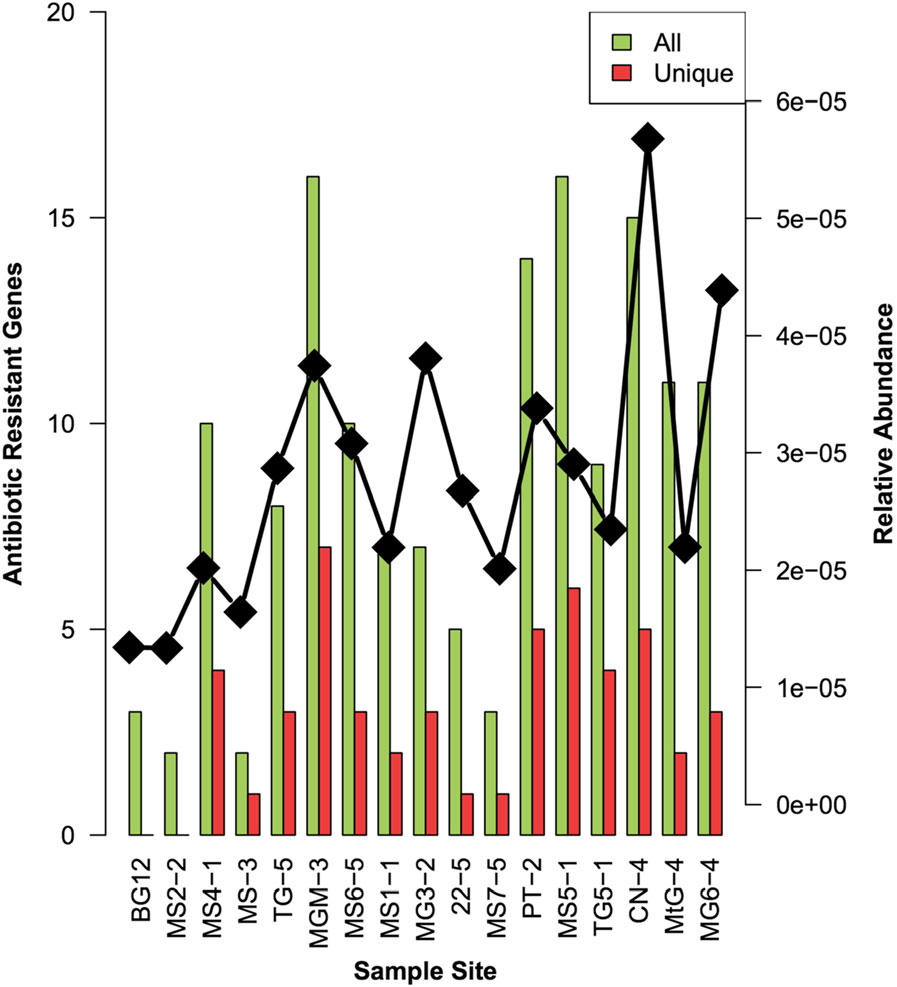
ARG frequencies across sampled sites. Number of different ARGs indicated in green, with the number of unique ARGs displayed in red, axis on the left. The relative ARG abundances are shown as a black line, axis on the right

In total, the identified ARGs spanned 23 ARG families (Table 1) and represent all known generic mechanisms of antibiotic resistance (resistance mutations, antibiotic efflux and antibiotic inactivation [7]). The distribution of ARGs was highly variable across the remote, pristine sites, ranging from 2 (sites MS2-2 and MS3-5) to 16 (sites MGM-3 and MS5-1) ARGs per site (Fig. 2). This variable dispersal extended to the frequency of unique ARGs per site, with MGM-3 (*n* = 7) and MS5-1 (*n* = 6) containing the most unique ARGs (Table 2). The relative abundance of ARGs was highest in sample CN-4 and the lowest in samples BG12-3 and MS2-2.

**Table 1:** ARG and ARG family frequencies and relative abundances found across the 17 sites

Sample site	Different ARGs	Unique ARGs	Total ARGs	Relative abundance^a^	Different ARG families	Unique ARG families	Total ARG families	Relative abundance^a^
BG12-3	3	0	3	1.34e−05	2	0	3	0.67
MS2-2	2	0	2	1.34e−05	2	0	2	1.0
MS4-1	10	4	12	2.02e−05	8	1	12	0.8
MS3-5	2	1	2	1.64e−05	2	0	2	1.0
TG1-5	8	3	8	2.87e−05	6	0	8	0.75
MGM-3	16	7	21	3.74e−05	11	1	21	0.6875
MS6-5	10	3	11	3.08e−05	5	0	11	0.5
MS1-1	7	2	7	2.19e−05	6	2	7	0.86
MG3-2	7	3	7	3.81e−05	6	0	7	0.86
MtG22-5	5	1	6	2.68e−05	4	0	6	0.8
MS7-5	3	1	3	2.01e−05	2	0	3	0.67
PT-2	14	5	20	3.38e−05	7	0	20	0.5
MS5-1	16	6	16	2.90e−05	10	0	16	0.63
TG5-1	9	4	9	2.35e−05	6	1	9	0.67
CN-4	15	5	18	5.68e−05	10	2	18	0.67
MtG-4	11	2	18	2.20e−05	5	0	18	0.45
MG6-4	11	3	14	4.38e−05	6	0	14	0.55

^a^Relative abundancy is calculated as the total number of ARG divided by the number of genes predicted per sampled site

**Table 2:** ARG families found exclusively in a single community

Sample site	ARG family description
MS4-1	Multidrug efflux pump
MGM-3	Dihydrofolate reductase, which cannot be inhibited by trimethoprim
MS1-1	Tet35 is a tetracycline efflux pump found in the Gram-negative *Vibrio* and *Stenotrophomonas*. Unrelated to other tet resistance genes
TG5-1	Aminoglycoside 6-N-acetyltransferase, which modifies aminoglycosides by acetylation
CN-4	Adenine transferase/methyltransferase, conferring resistance to erythromycin/kasugamycin
	Mutation frequency decline (Mfd) protein

We found a strong linear correlation between the number of unique ARGs and the number of unique taxa in each community (Pearson’s correlation, *r* = 0.89, *P* < 1.62e−06; Additional file 1: Figure S1). This trend supports a common feature in ecology, whereby higher levels of biodiversity are generally reflected by greater functional heterogeneity [52]. In contrast to other Antarctic regions, such as McMurdo Dry Valley soils [26] and glacial cores [21], we found a very diverse set of ARGs. This may be due to the sensitivity of our approach compared to functional screening, for example [10, 53]. An alternative reason for this difference could be due to antibiotic consumption by heterotrophic bacteria in these hyperoligotrophic soils (C and N concentrations were near accurate detection limits; Additional file 1: Table S1). Bacteria are known to be capable of surviving on a limited number of antibiotics, even using these inhibitory molecules as sole carbon sources [54], and a history of heterotrophic antibiotic degradation might minimise the pressure for ARG evolution.

**Fig. 3:**
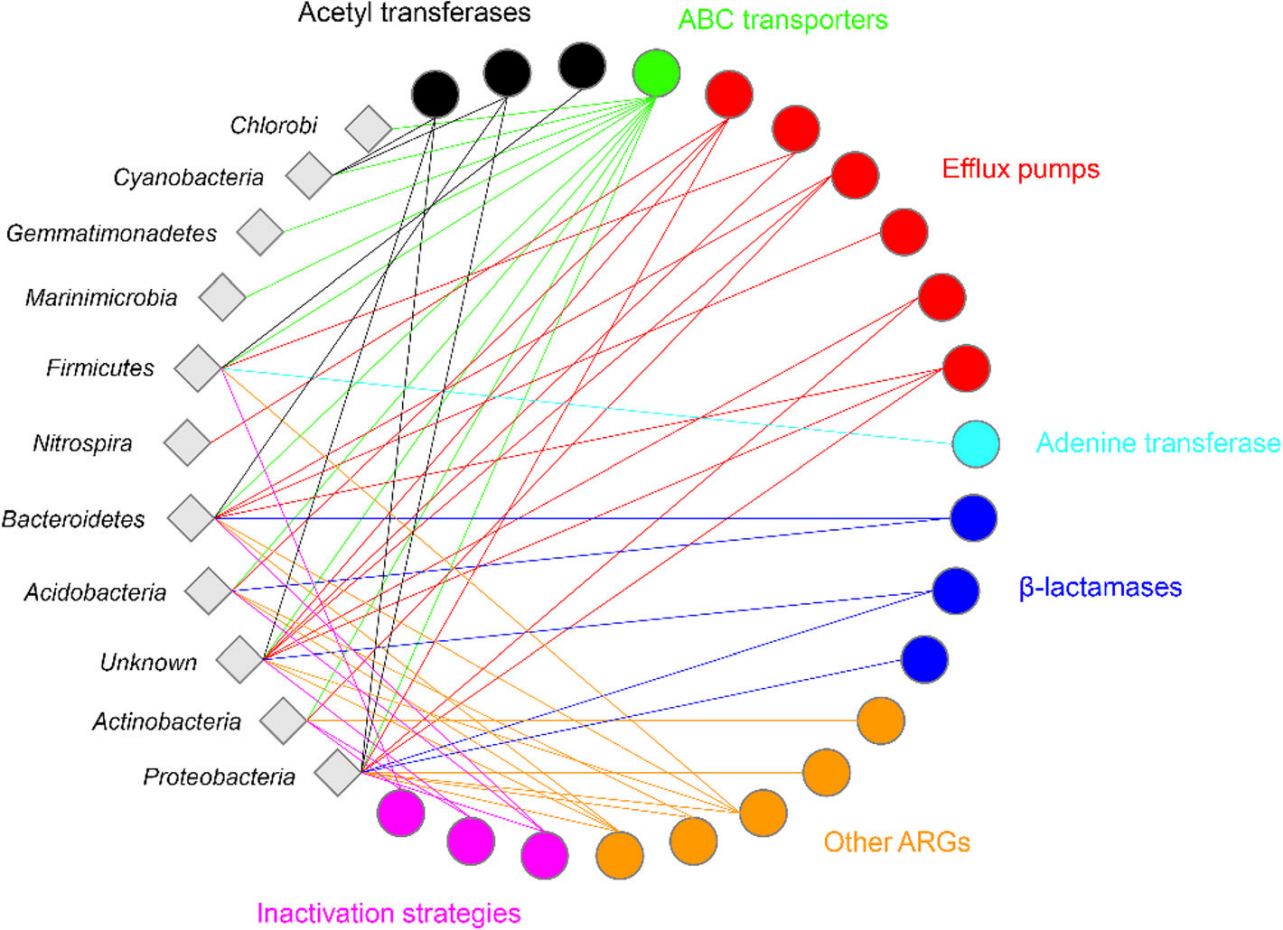
Co-occurrence network of ARG mechanisms showing resistance mechanisms encoded by diverse soil bacterial phyla. Phyla from all 17 soils that were assigned an ARG are presented here (diamond-shaped nodes), with significant co-occurrences with a specific ARG (circles) indicated (edges)

We predicted the source phylum of each ARG using comparisons to the entire NCBI protein non-redundant database (Fig. 3). Most ARGs (126 of 177; 71%) belonged to Gram-negative bacteria, while 35 ARGs (20%) could not be confidently classified beyond the kingdom level. A further 16 ARGs (9%) were assigned to Gram-positive bacteria, exclusively the *Bacilli* (*Firmicutes*) and *Actinobacteria*. The Gram-negative *Bacteroidetes* (41 ARGs) and *Acidobacteria* (37 ARGs) were the primary sources of ARGs across all sites. Together, these two phyla accounted for just under half of the ARGs found (78 out of 177) and both harboured all major antibiotic resistance strategies (Fig. 3). Bacterial phyla encoding multiple resistance mechanisms included *Proteobacteria* (30 ARGs), *Firmicutes* (16 ARGs), *Cyanobacteria* (8 ARGs) and *Actinobacteria* (5 ARGs). By contrast, *Nitrospira* (2 ARGs), *Chlorobi* (1 ARG), *Gemmatimonadetes* (1 ARG) and *Marinimicrobia* (1 ARG) contributed very few ARGs, which mirrored their low abundance within this environment [55].

Many clinical pathogens are members of the *Proteobacteria* [56]. *Proteobacteria* have been shown to harbour the greatest number of ARGs in different soil niches, reflecting their taxonomic dominance in those samples [26, 50, 54]. Similarly, the most abundant prokaryotic taxa in the Mackay Glacier soils, *Bacteroidetes* and *Acidobacteria*, contributed the most ARGs overall. This contrasts with the phylogenetic affiliations of ARGs in the human gut, for example, which were primarily assigned to abundant *Firmicutes* [57]. Overall, we support the contention that ARG distribution broadly reflects community membership [51]. In addition, our results serve to highlight the prevalence of ARGs in bacteria with large genomes (larger than 6 Mb) [54].

The two most abundant ARG families identified were undecaprenyl pyrophosphate phosphatases (UppP) and genes for efflux/transporter systems. We found that UppP genes, which confer resistance to Bacitracin, were assigned to the three most abundant bacterial phyla in these communities, *Bacteroidetes* (9 ARGs), *Acidobacteria* (7 ARGs) and *Proteobacteria* (3 ARGs), while a large proportion could not be assigned to a known taxonomic group (7 ARGs). These genes were present in 12 of the 17 metagenomes, suggesting that this is a relatively common mechanism of antibiotic resistance in soil communities, which extend to globally distributed soil biomes including forests, tundra and grassland ecosystems [50, 58].

Efflux transporters, which mediate the export of antibiotics across the cell surface and reduce intracellular antibiotic load [8], are some of the most common mechanisms of resistance in microorganisms [6]. ORFs annotated as efflux mechanisms were found in 13 of the 17 metagenomes, confirming that this is a common resistance strategy in soil bacterial populations. The most common transporter genes in these samples were the ancient super-family, adenosine triphosphate (ATP)-binding cassette (ABC) efflux/transporters [59]. We found a total of 50 ARGs assigned as ABC transporters (Table 3). ABC transporters couple ATP hydrolysis to solute efflux to actively transport compounds across the cell membrane [60]. We also found the *rosA* gene in many samples (19 ARGs), the product of which is an antiporter efflux pump. ARGs encoding macrolide transporter ATP-binding/permease proteins were some of the most common exporters and were present in 9 of the 17 soil resistomes. Tetracycline efflux pumps were uncommon (8 ARGs), and we found no other efflux pumps specific to a single class of antibiotics. Some communities possessed MDS (membrane-spanning domain) efflux pumps (2 ARGs) and multidrug efflux pumps (11 ARGs), which have the potential to remove a broad spectrum of antibiotics from the cell.

**Table 3:** ARGs and ARG families found in five or more of the communities

ARG description	No. of sites with ARG
Resistance-nodulation-cell division transporter system. Multidrug resistance efflux pump. Macrolide-specific efflux system	9
MacB is an ATP-binding cassette (ABC) transporter that exports macrolides with 14- or 15-membered lactones	8
dfrE is a chromosome-encoded dihydrofolate reductase found in *Enterococcus faecalis*	8
Efflux pump/potassium antiporter system. RosA: major facilitator superfamily transporter	7
Alanyl-tRNA synthetase conferring resistance to novobiocin in *Escherichia coli*	7
The enzymatic inactivation of rifampin by phosphorylation at the 21-OH position	6
Undecaprenyl pyrophosphate phosphatase, which consists in the sequestration of Undecaprenyl pyrophosphate	5
ARG family description	Number of sampled sites
Undecaprenyl pyrophosphate phosphatase, which consists in the sequestration of Undecaprenyl pyrophosphate	12
ABC efflux/transporter system	12
Efflux pump/potassium antiporter system. RosA: major facilitator superfamily transporter	9
dfrE is a chromosome-encoded dihydrofolate reductase found in *Enterococcus faecalis*	8
Aminocoumarin resistant alaS. Alanyl-tRNA synthetase conferring resistance to novobiocin in *Escherichia coli*	7
Resistance-nodulation-cell division transporter system. Multidrug resistance efflux pump	6
The enzymatic inactivation of rifampin by phosphorylation at the 21-OH position	6
Aminoglycoside acetyltransferase/nucleotidylyltransferase, which modifies aminoglycosides by acetylation/adenylylation	6
Major facilitator superfamily transporter, tetracycline efflux pump	5

Genes involved in antibiotic inactivation strategies were less common than either UppP genes or efflux pumps in the Antarctic soil metagenomes. Genes encoding β-lactamases, which confer resistance to β-lactam antibiotics by intracellular enzymatic degradation, are the most common antibiotic-inactivating ARGs in most microbial communities [2]. Surprisingly, we found a very low abundance of β-lactamases, despite identifying class A, B and C β-lactamase genes in a single sample (site MGM-3). This is in contrast with a recent study, based on GeoChip analysis, that reported a high diversity of β-lactamase genes in Antarctic McMurdo Dry Valley soil and lithic niches [26]. β-Lactamase genes are common components of the temperate soil resistome and have also recently been found at low levels in undisturbed cold soil ecosystems such as Arctic permafrost [11], glacial ice cores [21] and Alaskan soils [10, 61]. We note that the Mackay Glacier region soil samples used in our study come from much more remote sites than those of the McMurdo Dry Valley studies and are much less likely to have been exposed to anthropogenic input.

Interestingly, we found that rifampicin phosphorylation (8 ARGs) and erythromycin inactivation (1 ARG) were unique resistance mechanisms in Gram-positive bacteria, including members of the *Bacillus* spp. which are known to be resistant to aminoglycosides in the natural environment [11]. Gram-negative bacteria also harboured exclusive antibiotic inactivation mechanisms, such as genes for modifying naturally occurring antibiotics such as novobiocin (8 ARGs) and chloramphenicol (6 ARGs). Unexpectedly, we found a single ARG encoding resistance to trimethoprim, a synthetic antibiotic that inhibits DNA synthesis (Fig. 3). This finding supports the contention that aerosol transport within and to the Antarctic continent can result in the introduction of non-indigenous microorganisms [62, 63], serving as a mechanism for the introduction of antibiotic resistance genes into remote and pristine soil communities [5]. An alternative explanation for this finding could be the presence of thymidylate synthetase (*thyE*), which converts deoxyuridine monophosphate (dUMP) to deoxythymidine monophosphate (dTMP), at the 5′ end of the *drfA* sequence that encodes resistance to trimethoprim.

While we identified ARGs belonging to all the generic mechanisms of antibiotic resistance, we identified only 23 families of the 140 ARG families present in the concatenated *noradab* database, in all 17 metagenomes. Many of the ‘missing’ ARG families were those involved in the inactivation of synthetic antibiotics, such as Florfenicol and semi-synthetic derivatives such as Ciprofloxacin, although resistance genes for some natural antibiotics, such as Gentamycin, were not identified. We argue that the absence of ARGs targeting synthetic antibiotics reflects the pristine nature of the soil communities. This is entirely consistent with the observation that agricultural soil communities, that have been impacted by human and animal activities and high concentrations of antibiotics over relatively long periods, contain resistance genes for numerous synthetic antibiotics [48].

**Fig. 4:**
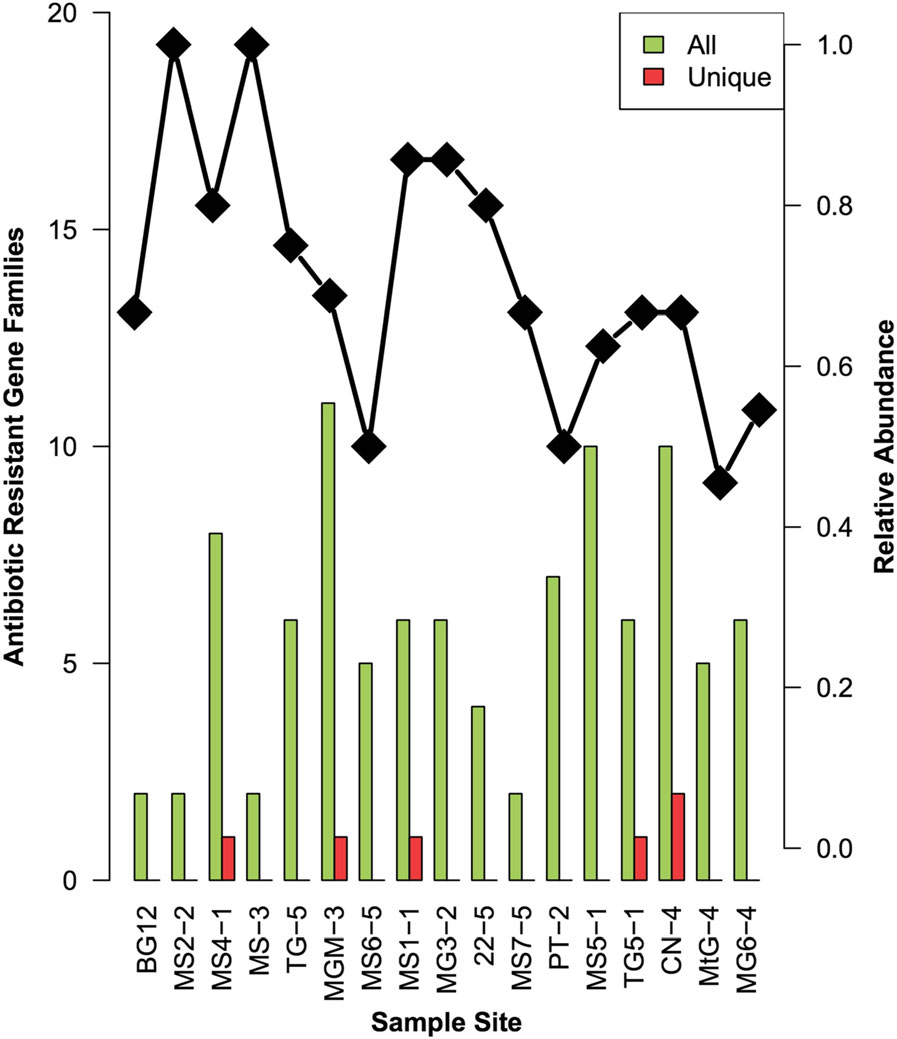
ARG family frequencies across sampled sites. The number of different ARG families are indicated in green, with the number of unique ARG families displayed in red, axis on the left. The black line represents the relative ARG family abundance, axis on the right

Our data showed substantial differences in AR families for different sample sites (Table 1, Fig. 4). The number of different ARG families in each community also varied considerably. For example, site MGM-3 contained a total of 11 different AR families, whereas MS2-2 and MS3-5 were the most ARG naïve, with only two ARG families in each community (Additional file 1: Figure S2). The number of unique ARG families per site did not exceed two, indicating shared resistance strategies across all communities. Overall, nine ARG families were present in five or more of the communities (Table 3). Only five of the sites contained a unique ARG family (Table 4).

**Table 4:** ARG host species frequencies in each community

Sample site	Different AR host species	Unique AR host species	Total AR host species	Total species	Relative abundance^a^
BG12-3	3	2	3	243	0.012
MS2-2	2	0	2	271	0.007
MS4-1	11	3	12	243	0.045
MS3-5	2	0	2	271	0.007
TG1-5	7	4	8	195	0.036
MGM-3	17	11	21	208	0.082
MS6-5	8	5	11	240	0.033
MS1-1	6	3	7	249	0.024
MG3-2	6	4	7	317	0.019
MtG22-5	6	4	6	216	0.028
MS7-5	2	0	3	241	0.008
PT-2	13	8	20	223	0.058
MS5-1	14	8	16	242	0.058
TG5-1	9	5	9	221	0.041
CN-4	15	8	18	194	0.077
MtG-4	9	4	18	210	0.043
MG6-4	10	2	14	229	0.044

^a^Relative abundance is calculated by dividing the number of different AR hosts by the number of different species found at each site

To explain the differences in ARG distribution between soil communities across the range of sample sites, we used a redundancy analysis (RDA) based on environmental physicochemical parameters (Additional file 1: Table S1). The diversity of ARGs was significantly driven by the percentage soil nitrogen (N) (*P* < 0.03; Additional file 1: Figure S3). No significant trends related to abiotic features were found for ARG families (data not shown), which may reflect the loss of discriminatory power at broader gene classification levels. Although most of the environmental factors measured here did not significantly influence the resistome family portfolio of individual sites, our observation that soil N influenced ARG composition in these hyperoligotrophic soils is consistent with studies showing significant differences in soil diversity and ARG composition resulting from N fertilisation [64, 65].

**Fig. 5:**
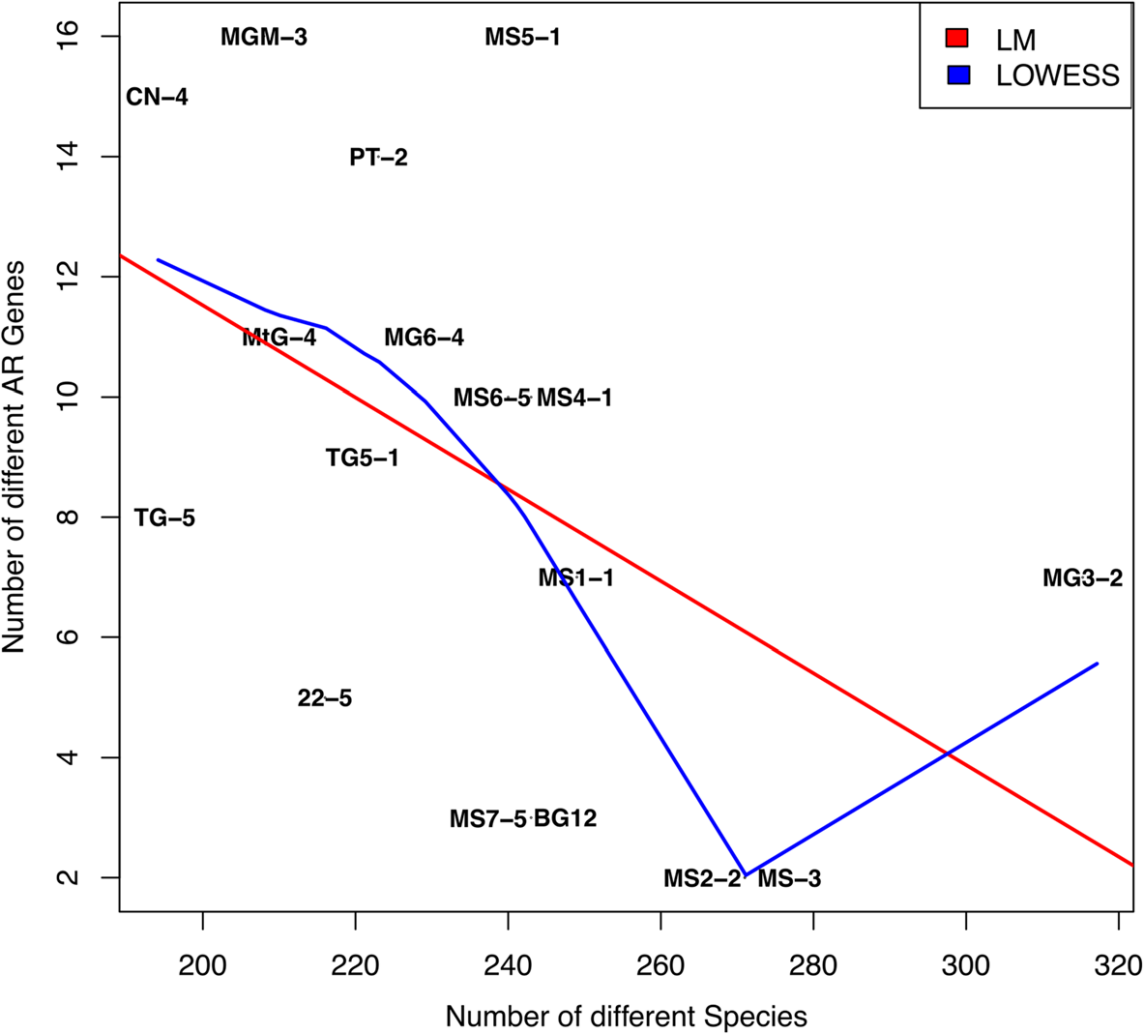
The number of ARGs and number of species per site. Linear model indicated in red and lowess in blue (Pearson’s correlation *r* = − 0.49, *P* < 0.05)

Strikingly, the number of ARGs per sample showed a significant negative correlation with the number of species per site (Pearson’s correlation; *r* = − 0.49, *P* < 0.05, Fig. 5). This is an interesting and novel result and might be attributed to the competitive exclusion of species by antibiotic exposure, potentially via the inhibition of the growth and activity of competing species [66]. This could favour the selection of pre-existing genotypes rather than leading to the development and acquisition of novel resistance mechanisms, as previously proposed [65]. However, if antibiotic concentrations do not reach levels of inhibition, as indicated in many soil environments [6], antibiotic production may instead serve to disrupt cellular signalling by acting as signal quenchers [67].

Interestingly, the contigs containing ARGs did not possess any flanking regions that shared similarities with the COG mobilomes categories. However, many contigs which did not possess an ARG were enriched with genes that shared similarities with a variety of transposons, phage, integrase and plasmid elements (*n* = 91,408). This result indicates that although contigs with ARGs lacked traces of mobile genetic elements, there is evidence of the capacity for substantial horizontal gene transfer in all 17 pristine Antarctic soil metagenomes, due to the presence of multiple mobilome-associated genes.

**Fig. 6:**
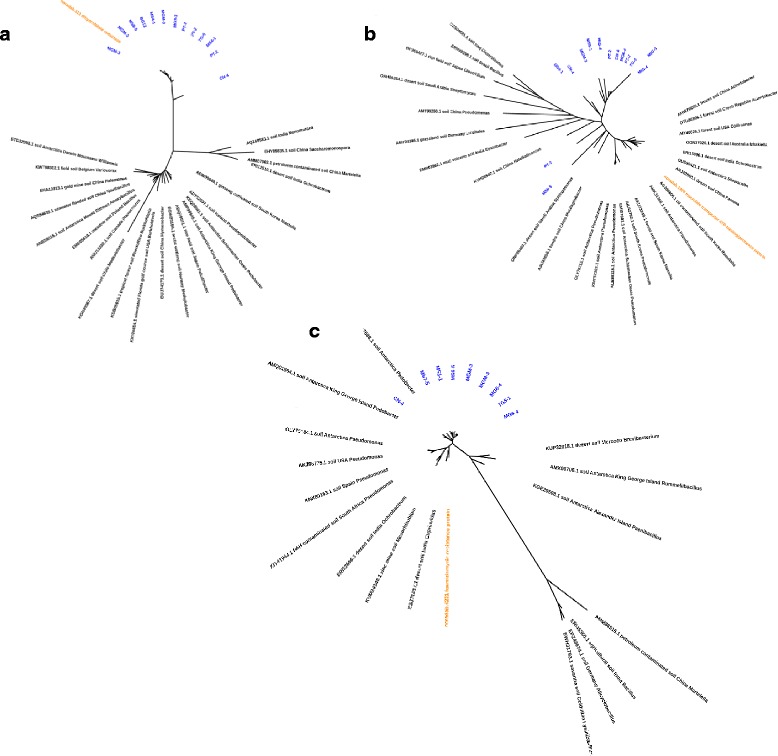
Unrooted Bayesian phylogeny of microbial antibiotic resistance gene sequences identified across the 17 Antarctic soil metagenomes. **a** Dihydrofolate reductase (*drfE*) genes, **b** Macrolide transporter ATP-binding permease proteins, and **c** major facilitator superfamily transporter sequences (*rosA*) are shown; blue denotes ORFs identified in the Antarctic metagenomes. Reference sequences are provided with accession numbers, and the protein sequence present in *noradab* is shown in orange

In order to shed light on the evolutionary history of the Antarctic soil resistome, we interrogated the phylogenetic placement of the three most abundant ARG classes, with respect to other environmental ARG sequences (Fig. 5), including cold and hot desert soil communities and many temperate soil biomes. Our analysis showed distinct clustering of our sequences from all other environmentally derived ARGs for all three resistance mechanisms, i.e. dihydrofolate reductase (*drfE*) genes (Fig. 6a), macrolide transporter ATP-binding permease proteins (Fig. 6b), and major facilitator superfamily transporter sequences (*rosA*) (Fig. 6c). Together, these results provide strong evidence that ARG homologues found in these remote and uncontaminated Antarctic soils represent legacy genes that were acquired, or evolved, in the distant past that over time formed part of the essential gene pools and have undergone niche-specific selective pressure. We argue that the low similarity to modern ARG variants could reflect either parallel evolutionary processes or the outward transport of historical ARGs as templates for subsequent evolution in more temperate environments. Phylogenetic analyses have placed the origins of some β-lactamase genes at over 2 billion years ago [2].

## Conclusions

Many studies have explored the distribution of antibiotic resistance in natural environments; however the spectrum of resistance determinants in remote pristine soil communities had not yet been elucidated. This is the first detailed metagenomic study of antibiotic resistance genes in remote, pristine soils that are naïve to anthropogenic antibiotic use. We have found 177 naturally occurring, historical genes conferring resistance to natural antibiotics. Our theory that most antibiotic resistance genes found here originate primarily from antibiotic-producing species was supported by the presence of antibiotic biosynthesis genes in many phyla encoding resistance and is further highlighted by the complete absence of mobile genetic elements (transposons, integrons and recombinases) flanking all putative ARGs found here. Antibiotic resistance appears to be transferred vertically over generations, with limited to no horizontal movement of ARGs between species. Thus, community members with antibiotic resistance may proliferate at the expense of susceptible counterparts [66]. This evidence supports the concept [65] that phylogeny, rather than HGT, drives differences in soil resistome content in the environment. This is entirely consistent with our finding that ARG abundance is negatively correlated with inferred species richness, indicating that communities with a high proportion of resistance elements exclude susceptible species, subsequently reducing soil diversity. Future challenges include understanding how both direct and indirect human-induced modulations influence the composition of these distinct soil communities, likely resulting in alterations to soil resistome properties.

## Additional file

Additional file 1: **Table S1.** Environmental factors of the 17 sampled sites. **Table S2.** The selected ARGs chosen from noradab, including the names, gene and ARG description, and ARG families. **Figure S1.** The number of unique ARGs and number of unique AR hosts per site. Linear model indicated in red and lowess in blue (Pearson’s correlation *r* = 0.89, *P* = 1.62e-06). **Figure S2.** ARG host frequencies across sampled sites. The number of different ARG hosts is indicated in green with the number of unique ARG hosts displayed in red, axis on the left. The black line represents the relative abundance, axis on the right. **Figure S3.** ARG redundancy analysis. The only environmental factor to display a significant impact was percentage N (*P* =0.024). (DOCX 129 kb)

## Abbreviations

ABC: Adenosine triphosphate (ATP)-binding cassette
ARBD: Antibiotic Resistance Genes Database
ARG: Antibiotic resistance gene
BG: Benson Glacier
CARD: Comprehensive Antibiotic Resistance Database
CN: Cliff Nunatak
HGT: Horizontal gene transfer
MG: Mackay Glacier
MGE: Mobile genetic elements
MGM: Mount George Murray
MS1–MS7: Mount Seuss
MtG: Mount Gran
Myr: Million years
*noradab*: Non-redundant antibiotic resistance database
PT: Pegtop Mountain
RDA: Redundancy analysis
TG: Towle Glacier
