# Supplementary material for: A reservoir of ‘historical’ antibiotic resistance genes in remote pristine Antarctic soils
Source: Microbiome. 2018 Feb 23;6:40. doi: 10.1186/s40168-018-0424-5 (PMC5824556; doi:10.1186/s40168-018-0424-5)

**Additional file for** **“A reservoir of ‘historical’ antibiotic resistance genes in remote pristine Antarctic soils”**

**Marc W. Van Goethem**, Rian Pierneef, Oliver K. I. Bezuidt, Yves Van De Peer, Don A. Cowan and Thulani P. Makhalanyane

**Table S1.** Environmental factors of the 17 sampled sites.

**Table S2.** The selected ARGs chosen from *noradab*, including the names, gene and ARG description, and ARG families.

**Figure S1.** The number of unique ARGs and number of unique AR hosts per site. Linear model indicated in red and lowess in blue (Pearson’s correlation *r* = 0.89, *P* = 1.62e-06).

**Figure S2.** ARG host frequencies across sampled sites. The number of different ARG hosts is indicated in green with the number of unique ARG hosts displayed in red, axis on the left. The black line represents the relative abundance, axis on the right.

**Figure S3.** ARG redundancy analysis. The only environmental factor to display a significant impact was percentage N (*P* = 0.024).

**Table S1.** Environmental factors of the 17 sampled sites.

| **Site** | **pH** | **P (ppm)** | **Na (cmol(+)/kg)** | **K (mg/kg)** | **Ca (cmol(+)/kg)** | **Mg (cmol(+)/kg)** | **% C** | **% N** | **Altitude (m.a.s.)** |
| --- | --- | --- | --- | --- | --- | --- | --- | --- | --- |
| BG12-3 | 8.20 | 11 | 0.6 | 61 | 2.16 | 0.89 | 0.1 | 0.02 | 1109 |
| MS2-2 | 7.79 | 12 | 1.4 | 26 | 6.33 | 1.41 | 0.12 | 0.016 | 497 |
| MS4-1 | 7.74 | 20 | 0.37 | 299 | 6.43 | 1.02 | 0.12 | 0.021 | 527 |
| MS3-5 | 7.81 | 9 | 0.7 | 35 | 6.04 | 1.33 | 0.1 | 0.017 | 547 |
| TG1-5 | 8.14 | 18 | 0.07 | 83 | 2.8 | 1.62 | 0.12 | 0.028 | 1017 |
| MGM-3 | 7.65 | 25 | 0.34 | 32 | 1.0 | 0.47 | 0.12 | 0.032 | 157 |
| MS6-5 | 8.70 | 22 | 0.94 | 116 | 4.95 | 0.91 | 0.12 | 0.017 | 488 |
| MS1-1 | 7.58 | 24 | 1.92 | 78 | 12.78 | 2.1 | 0.15 | 0.032 | 556 |
| MG3-2 | 7.53 | 14 | 0.13 | 58 | 1.29 | 0.78 | 0.1 | 0.02 | 189 |
| MtG22-5 | 7.94 | 17 | 0.32 | 56 | 4.48 | 1.37 | 0.1 | 0.028 | 963 |
| MS7-5 | 7.94 | 60 | 0.53 | 54 | 7.97 | 2.15 | 0.13 | 0.026 | 440 |
| PT-2 | 8.21 | 15 | 0.07 | 44 | 1.09 | 0.53 | 0.12 | 0.008 | 811 |
| MS5-1 | 7.77 | 23 | 0.49 | 87 | 4.02 | 0.98 | 0.1 | 0.01 | 576 |
| TG5-1 | 8.08 | 15 | 0.28 | 22 | 2.97 | 1.57 | 0.12 | 0.019 | 729 |
| CN-4 | 7.95 | 19 | 0.07 | 75 | 0.48 | 0.32 | 0.14 | 0.016 | 220 |
| MtG-4 | 8.17 | 4 | 0.13 | 15 | 3.7 | 0.44 | 0.1 | 0.024 | 652 |
| MG6-4 | 7.86 | 12 | 0.33 | 29 | 1.49 | 0.63 | 0.12 | 0.016 | 763 |

**Table S2.** The selected ARGs chosen from *noradab*, including the names, gene and ARG description, and ARG families.

| **ARGs** | **Gene Description** | **ARG Description** | **ARG Family** |
| --- | --- | --- | --- |
| noradab.113 | Dihydrofolate reductase | dfrE is a chromosome-encoded dihydrofolate reductase found in *Enterococcus faecalis* | dfrE is a chromosome-encoded dihydrofolate reductase found in *Enterococcus faecalis*. |
| noradab.1928 | Macrolide transporter ATP-binding/permease protein | Resistance-nodulation-cell division transporter system. | ABC efflux/transporter system. |
| noradab.4221 | Fosmidomycin resistance protein | Efflux pump/potassium antiporter system | Efflux pump/potassium antiporter system. RosA: Major facilitator superfamily transporter. |

**Figure S1.** The number of unique ARGs and number of unique AR hosts per site. Linear model indicated in red and lowess in blue (Pearson’s correlation *r* = 0.89, *P* = 1.62e-06).


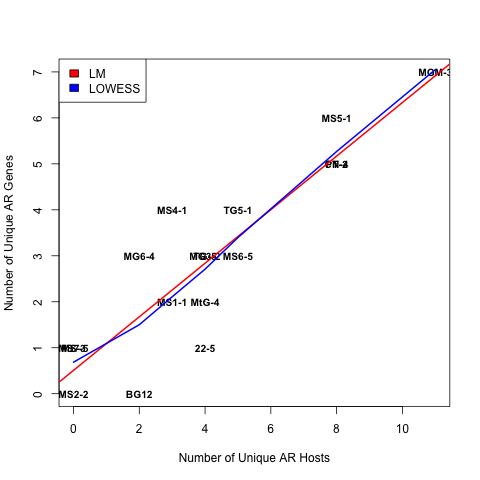


**Figure S2.** ARG host frequencies across sampled sites. The number of different ARG hosts is indicated in green with the number of unique ARG hosts displayed in red, axis on the left. The black line represents the relative abundance, axis on the right.


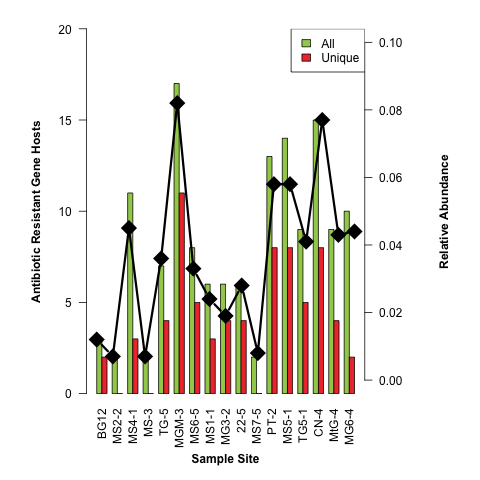


**Figure S3.** ARG redundancy analysis. The only environmental factor to display a significant impact was percentage N (*P* = 0.024).


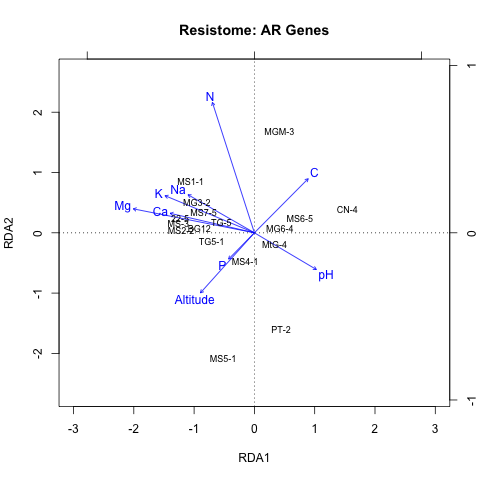

Supplement: Supplementary file 1 — Table S1. Environmental factors of the 17 sampled sites. Table S2. The selected ARGs chosen from noradab, including the names, gene and ARG description, and ARG families. Figure S1. The number of unique ARGs and number of unique AR hosts per site. Linear model indicated in red and lowess in blue (Pearson’s correlation r = 0.89, P = 1.62e-06). Figure S2. ARG host frequencies across sampled sites. The number of different ARG hosts is indicated in green with the number of unique ARG hosts displayed in red, axis on the left. The black line represents the relative abundance, axis on the right. Figure S3. ARG redundancy analysis. The only environmental factor to display a significant impact was percentage N (P =0.024). (DOCX 129 kb) [file 40168_2018_424_MOESM1_ESM.docx]
